# Manufacture of High-Performance Tidal Turbine Blades Using Advanced Composite Manufacturing Technologies

**DOI:** 10.1007/s10443-021-09967-y

**Published:** 2021-09-16

**Authors:** William Finnegan, Ronan Allen, Conor Glennon, James Maguire, Michael Flanagan, Tomas Flanagan

**Affiliations:** 1 Civil Engineering, School of Engineering, National University of Ireland Galway, Galway, Ireland; 2 MaREI Centre, Ryan Institute, National University of Ireland Galway, Galway, Ireland; 3 ÉireComposites Teo, An Choill Rua, Inverin, Co., Galway, Ireland; 4 School of Engineering, Institute for Materials and Processes, The University of Edinburgh, Edinburgh, UK

**Keywords:** Blades, Composite materials, Mechanical testing, Structural design, Tidal energy

## Abstract

After wind and solar energy, tidal energy presents the most prominent opportunity for generating energy from renewable sources. However, due to the harsh environment that tidal turbines are deployed in, a number of design and manufacture challenges are presented to engineers. As a consequence of the harsh environment, the loadings on the turbine blades are much greater than that on wind turbine blades and, therefore, require advanced solutions to be able to survive in this environment. In order to avoid issues with corrosion, tidal turbine blades are mainly manufactured from fibre reinforced polymer composite material. As a result, the main design and manufacture challenges are related to the main structural aspects of the blade, which are the spar and root, and the connection between the blade and the turbine hub. Therefore, in this paper, a range of advanced manufacturing technologies for producing a 1 MW tidal turbine blade are developed. The main novelty in this study comes with the challenges that are overcome due to the size of the blade, resulting in thickness composite sections (> 130 mm in places), the fast changes in geometry over a short length that isn’t the case for wind blades and the required durability of the material in the marine environment. These advances aim to increase the likelihood of survival of tidal turbine blades in operation for a design life of 20 + years.

## Introduction

As the global tidal stream energy sector moves closer to commercial viability, additional challenges are presented as developers strive to lower the levelised cost of energy in order to challenge the low cost associated with generating energy from fossil fuels. In order to achieve greater economic and sustainability targets, each key component of a tidal turbine needs to be designed, manufactured and operated as efficiently as possible. One of the key components for many tidal energy converters is the turbine blades, whether they are vertically, horizontally or otherwise orientated. During operation, these blades encounter high, variable loading conditions, including impact loadings, while being constantly submerged in water.

In 2019, the installed capacity of tidal stream energy in Europe reached 27.7 MW [1], which is almost four times as much as the rest of the world. While the total electricity produced in Europe from tidal energy increased by 15 GWh to a total to date of 49 GWh [1]. A key material technology that can be used to produce tidal turbine blades that won’t corrode in these submerged operational conditions is fibre reinforced polymers – for example, glass fibre reinforced epoxy. However, the use of this material presents the developer with an additional challenge of a reduction in performance in terms of tensile and compressive strength due to water ingression, which needs to be accounted for in the design and manufacture stage of blade production. The high, variable loadings on tidal turbine blades cause high bending moment and shear loads, which need to be allowed for within the structural design of the supporting sections of the blade. For horizontal axis tidal turbine blades, these sections are primarily the spar, which runs the length of the blade, and the root connection. Robust connections between these two sections and at the root, along with thick section composite structures, are often used to withstand these high forces and moments. When designing these key structural components, advanced numerical models have been developed for tidal turbine blades [2–4], which includes damage prediction modelling [5] and the effect of the environment on mechanical performance of the blade materials [6, 7].

In order to ensure the longevity of the blade throughout the desired design life, the root connection must withstand the operational loadings and survive the harshest of conditions. This is due to the fact that the blade weight, along with all of the static and fatigue loads and the resulting bending moments, are supported at the root of the blade. This results in the need for a highly robust root connection. Currently, there are a number of solutions available, which are installed after the blade is cured. However, issues arise with bonding to the composite materials and with dislodgement during installation. Therefore, a solution that presents a robust connection that can be installed within the main manufacturing stages of the blade is required.

Previously, there has been a limited amount of publications on the manufacture of thick section composite structures. However, Vaidya et al. [8] presented the processes required for the production of thick-section composite parts using cost-effective liquid moulding processes, such as resin transfer moulding (RTM) and vacuum assisted resin transfer/infusion moulding (VARTM). Also, Maguire et al. [9] described the manufacturing process in detail as they characterised epoxy powders for processing thick section composite structures, including a description of the relationship between glass transition temperature and the degree-of-cure. Additionally, Maguire et al. [10] used finite element software to model the resin transfer during the manufacture of thick section parts with low-cost fibre reinforced polymers, and Matveev et al. [11] performed a numerical study to investigate the required processing conditions of thick section composite structures, in order to analyse their effect on consolidation of thick composite components. For wind turbine blades, the most commonly used connection between the blade root and rotor hub is the T-bolt attachment. The standard DNVGL–SE-0164 [12] provides principles, technical requirements and guidance for the design, construction and in-service inspection of tidal turbines, including the design and manufacture and testing of tidal blades including a section detailing the requirements at the root connection. Additionally, the specific certification requirements and respective deliverables for tidal turbines are specified in DNVGL–SE-0163 [13] for the certification of tidal turbine blades.

**Fig. 1:**
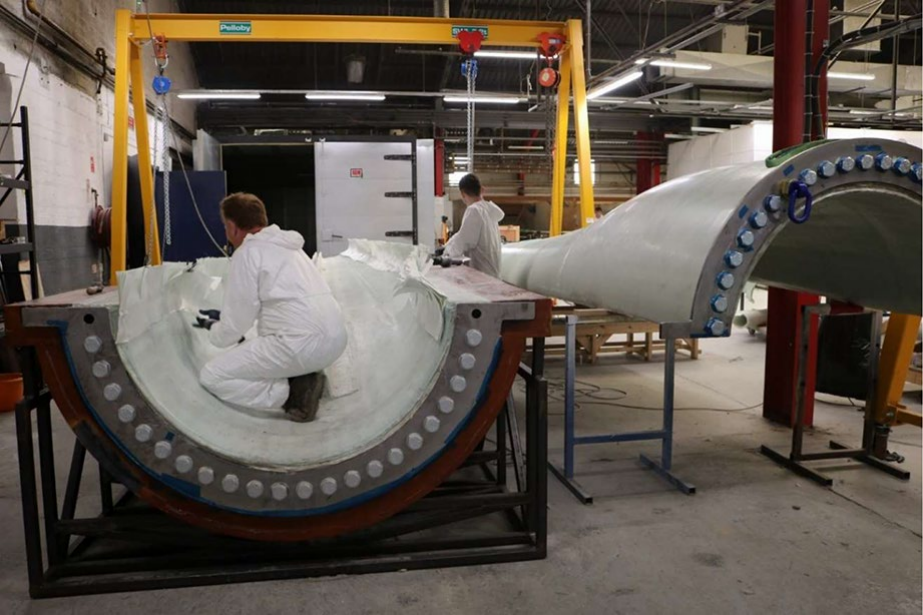
Full-scale tidal turbine blade during manufacture that is detailed in this study

In this paper, a range of advanced manufacturing technologies for producing a large full-scale tidal turbine blade are developed. Previously, some of these concepts have been introduced in Glennon et al. [14] without much detail, prior to the manufacture of the full-scale tidal turbine blade. Initially, the findings from a comprehensive study on the effect of saturated conditions on the material properties of glass fibre reinforced powder epoxy composite material, which is being used to manufacture the tidal turbine blade, along with using a cured bonding technique instead of adhesive bonding, are discussed. The key technologies being developed are thick section laminate manufacture for the root and spar caps and an innovative root connection system. These technologies have been tested on a sub-component level and the results are presented. Following on from these successful trials, a full-scale tidal turbine blade, which incorporates these advanced manufacturing technologies, has been produced, which is shown during manufacture in Fig. 1. The blade has a total length of 8 m and is part of a 2-blade 1 MW rotor. The novelty in this study comes with the challenges that are overcome due to the size of the blade, which isn’t the case for small scale trials, which includes the thickness of the composite sections at the root and along the spar caps (> 130 mm in places), the fast changes in geometry over a short length that isn’t the case for wind blades and the required durability of the material in the marine environment. The next stage of development is to undergo a rigorous structural testing programme in order to further de-risk the blade design and manufacture.

## Materials and Methods

### Aim and Objectives

The overarching aim of this study is to develop advanced technologies for manufacturing tidal turbine blades in a cost-effective and efficient manner. Since tidal energy is still an emerging market, it is vital that the levelised cost of tidal energy is reduced to a level that is competitive with energy from fossil fuels and achieving the proposed aim will contribute to this. However, in order to achieve the overarching aim of this study, the completion of the following objectives is necessary:

- To determine the material properties of the blade substrate material in both dry and saturated conditions, which are representative of the operational conditions of the blade.
- To develop manufacturing and processing technologies to successfully manufacture thick section composite structures.
- To perform tensile and fatigue testing to de-risk a more efficient design for a robust root connection to attach the thick section root part of the blade to the rotor hub.
- To develop additional manufacturing processes that are essential in producing tidal turbine blades.
- To trial the new technologies through the manufacture of a full-scale tidal turbine blade (shown during manufacture in Fig. 1).

Once the full-scale tidal turbine blade has been manufactured, it will be exposed to a rigorous structural testing programme, which includes high-load static, dynamic and fatigue tests that replicate the operational conditions of the turbine, in order to further de-risk the blade design and manufacture.

### Methodology

**Fig. 2:**
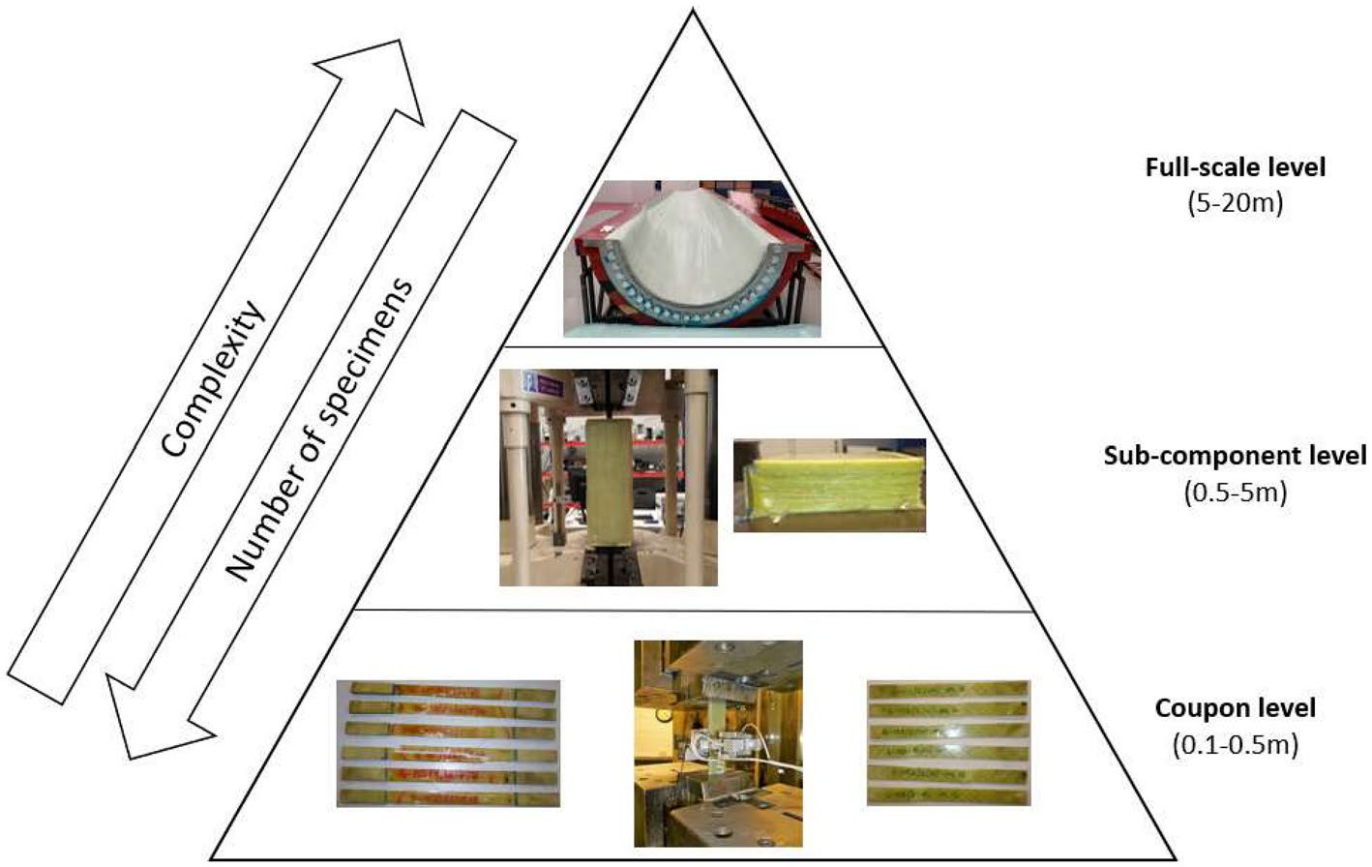
Pyramid concept applied to the development of manufacturing processes for a full-scale tidal turbine blade that is used in this study

In this paper, a range of advanced manufacturing processes for a full-scale tidal turbine blade are being developed. The analysis used in this study is based on the same pyramid concept that is used by Lopes et al. [15], which is summarised graphically in Fig. 2. The pyramid concept breaks up the development into three distinct sections, which have increasing size and complexity with a decrease in the number of specimens manufactured and tested, as follows:

- Coupon level trials – static and fatigue testing of material specimens, which is described in detail in Sect. 3.
- Sub-component level trials – manufacturing demonstrators, including thick-section laminates and root connections, are trialled and tested in Sect. 4.
- Full-scale blade manufacture – the advanced manufacturing processes being developed in this study are used to manufacture the full-scale tidal turbine blade in Sect. 5.

### Materials

The primary material that is used to manufacture the tidal turbine blade in this study is the composite material glass fibre reinforced powder epoxy, which is based on ÉireComposites’ Composites Powder Epoxy Technology, or CPET. CPET has been presented previously in [9, 16, 17] and has a number of advantages over traditional composite materials, including small through-thickness wet out requirement, good fibre volume fraction control at material manufacture stage, low exotherm during cure and it requires a vacuum bag only, out-of-autoclave cure. Additionally, the raw material can be stored at ambient temperatures and has a very long shelf life, when compared to tradition epoxy resins. Unidirectional glass fibre reinforced CPET is used in the manufacture the main structural elements of the tidal turbine blade, which are the spar caps and web, and, therefore, particular attention is paid to investigating its material properties in saturated conditions, which is detailed in Sect. 3.

The root connections, which are discussed in detail in Sect. 4.2, are made from S355 grade steel, which is a medium tensile, low carbon manganese steel. These are embedded into the root of the tidal turbine blade during the composite material lay-up and bonded within the blade during the curing.

## Coupon-Scale Experimental Trials

Unidirectional glass fibre reinforced CPET is used in the manufacture of the main structural elements of the tidal turbine blade, which are the spar caps and web. As tidal turbine blades are immersed underwater for the duration of the operational life, it is necessary to investigate the effect of water immersion on unidirectional glass fibre reinforced CPET. Therefore, mechanical testing was performed on specimens that were not immersed as a baseline and a set of specimens that were immersed in order to investigate if there is any change in their mechanical properties. It should be noted that no water ingress protection measures were applied to the specimens during this study.

### Water Immersion of CPET Specimens

**Fig. 3:**
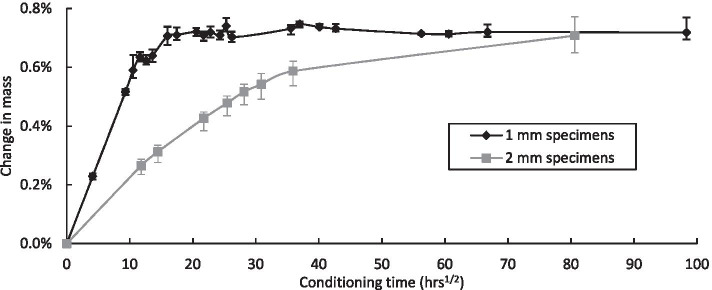
Change in mass of traveller specimens of 1 mm and 2 mm thick over a period of 400 days

Similar to a number of previous studies (Grogan et al. [18] and Walls et al. [6]), test specimens were placed in an water conditioning tank at 50 °C, in order to accelerate the aging of the composite material over the period of the testing programme. Along with the test specimens, two sets of traveller specimens, which were 25 × 25 mm and either 1 mm or 2 mm in thickness, were also immersed in the water conditioning tank in order to monitor water absorption of the composite material by recording the change in mass of the specimens. The traveller specimens are so called because they physically travel with the larger tensile test specimens and are weighed regularly using a high precision weighing scales, which is accurate to 0·0001 g. Both the traveller specimens and the test specimens have machined surfaces that are exposed to the water, which could influence the moisture uptake. Before weighing, the traveller specimens were carefully wiped with a lint-free cloth and allowed to cool to ambient temperature, which is a controlled environment at 23 °C and 50% relative humidity. The change in mass, which signifies the water absorption, for the two sets of traveller specimens over a 400-day period is shown in Fig. 3. It is evident from this figure that water absorption occurs at a much quicker rate for the thinner 1 mm specimens, compared to the 2 mm specimens. The 1 mm specimens achieved an average change in mass of 0.7% by Day 11, where the maximum average change in mass is 0.718% on Day 400, while this rate wasn’t reached by the 2 mm specimens until Day 55 at the earliest. Change in mass for the 2 mm specimens wasn’t recorded between Day 54 and Day 273, at which point the average change in mass is 0.708%.

### Static Testing Programme

**Fig. 4:**
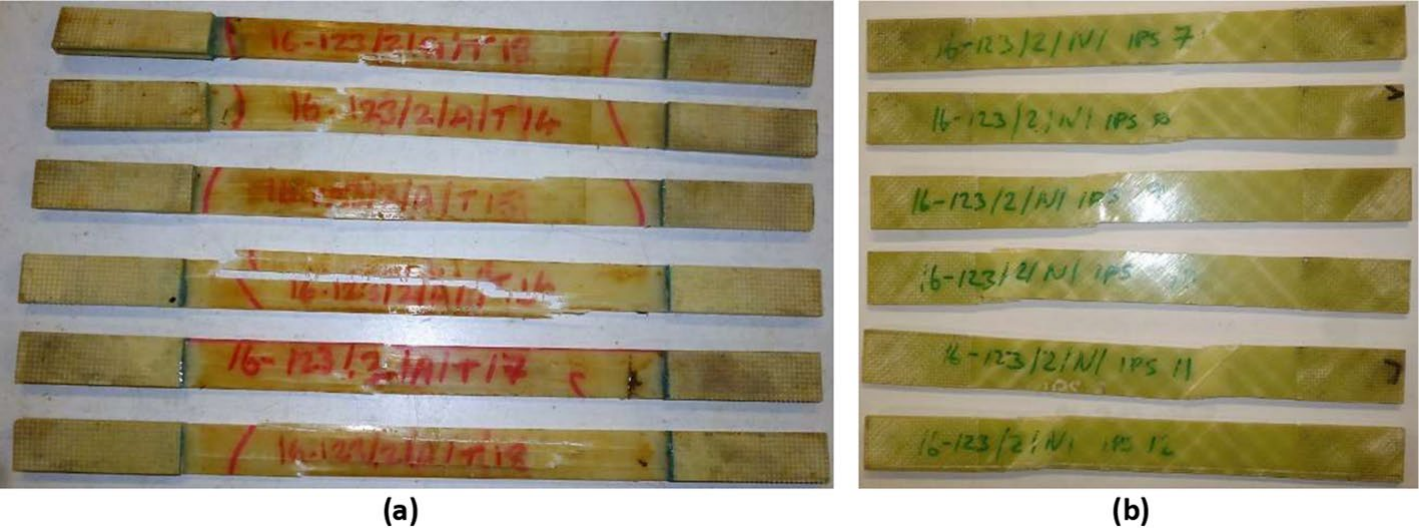
A selection of the specimens used for the mechanical testing programmes for (**a**) the tension tests and (**b**) the in-plane shear strength tests

The static testing programme was performed in accordance to ASTM D3039/D3039M-17 [19], which is the standard test method for tensile properties of polymer matrix composite materials, and also adheres to the relevant ISO standards. The static tests were conducted in a controlled environment at ambient temperature and relative humidity of 23 °C and 50%, respectively. The mechanical tests performed on the unidirectional glass fibre reinforced CPET specimens, include 0° and 90° static tension tests to ISO 527 and in-plane shear tests to ISO 14,129. Initially, a set of test specimens that were not immersed were tested as a baseline. Following this, two sets of test specimens, which were immersed in a water conditioning tank at 50 °C for approximately 60 days and 183 days, were tested in order to investigate any effect that the water immersion had on the composite material. A selection of the specimens used for the mechanical testing programmes are shown in Fig. 4, where specimens for the tension tests are shown in Fig. 4a and specimens for the in-plane shear strength tests are shown in Fig. 4b.

**Fig. 5:**
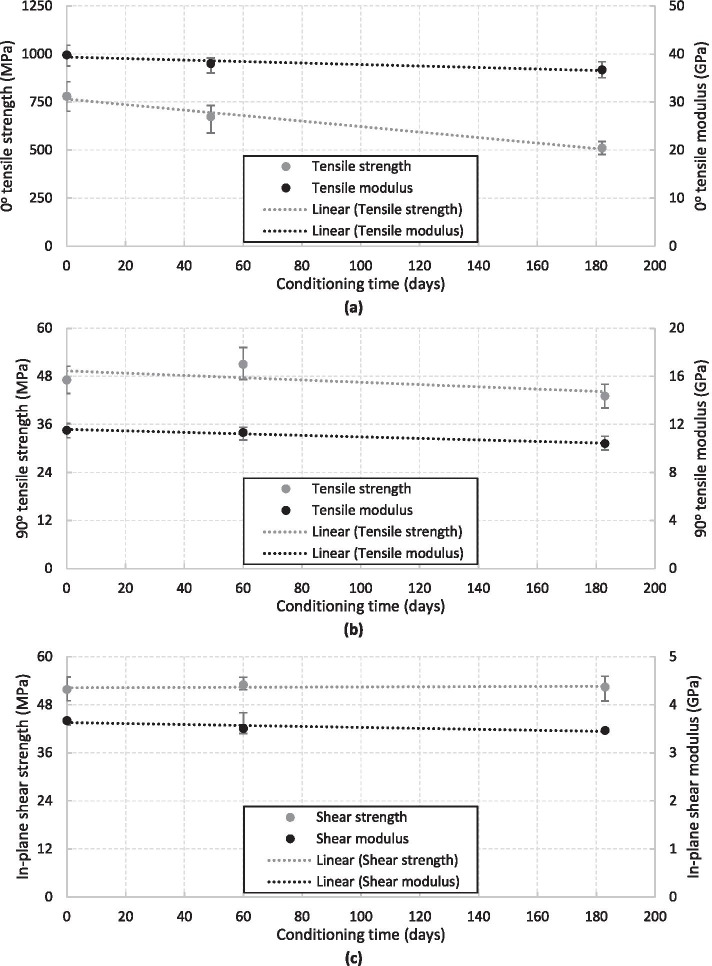
Results from the static testing programme, detailing (**a**) the 0° tensile properties, (**b**) the 90° tensile properties and (**c**) the in-plane shear properties

The results from the three mechanical static tests performed on the unidirectional glass fibre reinforced CPET specimens are given in Table 1 and shown graphically in Fig. 5. The test specimens used had an average fibre volume fraction of 50% and the actual data, rather than it being normalised, has been presented. For the 0° tension test, there is a knockdown in the tensile strength and tensile modulus of approximately 35% and 8%, respectively, after 182 days of conditioning in water at 50 °C, which can be seen in Fig. 5a. As the 0° direction is in line with the fibres, it is the preferred loading direction for the composite material when in operation. Therefore, this reduction in strength needs to be incorporated into the design of a marine composite structure. There is less of a reduction observed for the 90° tension testing of the specimens, where a knockdown in the tensile strength and tensile modulus of approximately 9% and 10%, respectively, was observed, after 183 days of conditioning in water at 50 °C, which can be seen in Fig. 5b. Again, this reduction will need to be incorporated into the design of a marine composite structure. However, there was very little effect observed for the in-plane shear testing of the specimens, where there was an insignificant (1%) change in the shear strength and a 6% knockdown in the shear modulus over the 183 days of conditioning in water at 50 °C, which can be seen in Fig. 5c.

**Table 1:** Summary of the results from the static testing programme

**Testing programme**	**Conditioning time (days)**	**No. of specimens**	**Strength (MPa)**^**1**^	**Modulus (GPa)**^**a**^
0° tension	0	12	780.5 (702.3–855.9)	39.8 (37.5–41.8)
	49	6	674.5 (589.2–733.3)	38 (36–39.2)
	182	6	510.8 (477.2–544.4)	36.7 (35–38.4)
90° tension	0	18	47.1 (43.7–50.5)	11.5 (10.9–12.1)
	60	12	51 (47.2–55.2)	11.3 (10.7–11.8)
	183	6	43.1 (40.1–46)	10.4 (9.9–11)
In-plane shear	0	12	51.8 (49–54.9)	3.7 (3.6–3.7)
	60	6	53 (51.7–54.9)	3.5 (3.4–3.8)
	183	6	52.4 (48.9–55.1)	3.5 (3.4–3.5)

^a^Displayed as ‘average (range)’

### Fatigue Testing Programme

**Fig. 6:**
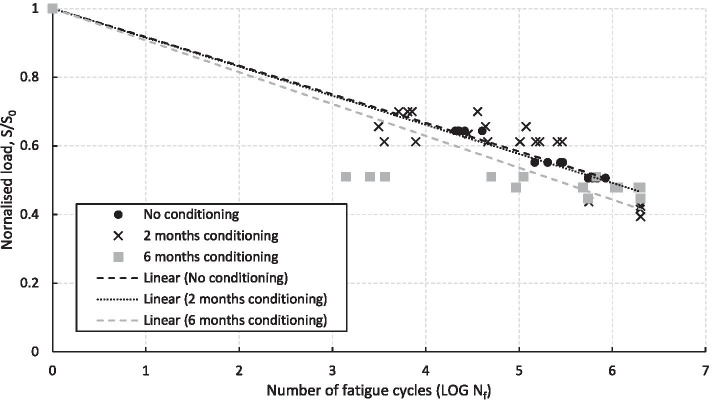
Results from the fatigue testing programme

The fatigue testing programme was performed in accordance to ASTM D3479/D3479M-19 [20], which is the standard test method for tension-tension fatigue of polymer matrix composite materials, where the fatigue tests were, again, conducted in a controlled environment at ambient temperature and relative humidity of 23 °C and 50%, respectively. Initially, fatigue testing of 13 dry specimens, who did not undergo any conditioning, were completed as a baseline. After 2 months (60 days) of conditioning, 27 specimens were tested in fatigue and a slight knock-down on average compared to the baseline case is observed. After 6 months (180 days) of conditioning, a further 18 specimens were tested in fatigue, where a knock-down on average compared to the baseline case and the 2 months conditioning is observed. Therefore, demonstrating there is an effect on the material when exposed to a fatigue loading. As this was the longest conditioning period, any inconsistencies in the material and preparation of coupons would become more apparent, resulting in the greater scatter in the data, which is observed in Fig. 6. This is due to greater water ingress through the laminates due to the amount of end fibres exposed as a result of a number of factors, including off-square cutting of specimens and tabbing inconsistencies that uses adhesives that effects the amount of end fibres exposed. Also, as the greatest knock-down in performance was expected, the highest normalised load applied was of S/S_0_ = 0.51 as it was anticipated that higher loads would result in too few testing cycles completed before failure. A summary of the results from the fatigue testing programme is presented in Table 2 and graphically in Fig. 6, where the average knock-down due to conditioning has also been included.

**Table 2:** Summary of the results from the fatigue testing programme

**Conditioning time (days)**	**No. of specimens**	**Normalised load, S/S**_**0**_	**No. of fatigue cycles**^**a**^**, N**_**f**_
0	5	0.644	26,461 (20,710–40,212)
0	4	0.552	230,426 (146,691–294,702)
0	4	0.506	675,445 (550,187–842,463)
60	4	0.7	13,672 (5,116–36,037)
60	3	0.656	55,191 (3,118–118,769)
60	8	0.612	127,986 (3,568–290,528)
60	6	0.394	1,758,462 (562,533–2,000,000)
180	6	0.51	140,191 (1,394–671,102)
180	6	0.478	1,116,413 (92,633–2,000,000)
180	6	0.447	1,758,396 (550,378–2,000,000)

^a^Displayed as ‘average (range)’

### Lap Shear Testing Programme

One of the key advantages of using the CPET technology is that the turbine blade can be made in subcomponents, by b-stage curing (at 50 °C) to form a solid part, and then assembled, where it undergoes a final cure at 180 °C. This composites manufacturing method is known as the ‘One-shot manufacturing process’ and is described in greater detail in Sect. 5.1. Using this manufacturing method, joints between subcomponents are bonded together during the cure, which negates the need for adhesives.

Therefore, in this section, a lap shear testing programme has been performed to investigate the performance of the cured bond using CPET instead of adhesive bonding. Test specimens were produced from a 2 mm glass fibre CPET panel with a 2-ply unidirectional fibre lay up of [0]_2_, where each specimen was 25 mm wide, and three manufacturing methods were used to create the bond at the overlap, which are summarised as follows:

- Adhesive bonding
- B-staged and then fully cured
- Fully cured and then fully cured again (cured-cured)

The lap shear testing programme was carried out in accordance with EN 2243 using a 250 kN capacity Zwick test machine. In order to fix the test specimens in place, hydraulic wedge grips were used, and the load was applied at 1.3 mm/min until failure. An extensometer mounted at the centre of the specimen was used to record the strains. It should be noted that the test specimens were not tabbed, as per test standard.

A summary of the testing for the three manufacturing methods are presented in Table 3. A total of 12 specimens were bonded using an adhesive and found that the adhesive bond has an average lap shear strength of 27.1 MPa. 15 specimens that were manufactured using b-staging followed by a full cure were tested and found to have a much higher average lap shear strength (43.7 MPa) than the adhesive bond. This was also the case for the 14 specimens that were manufactured using the cured-cured approach, which have an average lap shear strength of 41 MPa. It is evident from this testing programme that curing the assembled subcomponents provides much stronger bonded joints, when compared to the traditional adhesive bonding approach. Therefore, this method will be used in manufacturing the full-scale tidal turbine blade, which is detailed in Sect. 5.

**Table 3:** Summary of the results from the lap shear testing programme

**Method**	**No. of specimens**	**Average overlap (mm)**	**Average specimen length (mm)**	**Lap shear strength (MPa)**
Adhesive	12	13.27	188.85	27.12 (20.47–33.57)
B-staged then cured	15	13.08	192.82	43.7 (40.85–46.36)
Cured-cured	14	12.83	191.53	41.05 (35.08–44.67)

### Coupon-Scale Experimental Trials Discussion

During this study, the saturation of the composite material was accelerated by increasing the temperature of the water to 50 °C. Based on the study by Kennedy et al. [21], a conservative estimate on the acceleration of the aging process is a factor of 10 at 30 °C, which would lead to a factor of 101 for conditioning at 50 °C, assuming an operational temperature of 12 °C. Therefore, for the conditioning time of 183 days that took place in this study, the equivalent submerged operational time would be close to 50 years, which is well in excess of a typical design life for a tidal turbine of 25 years. The maximum absorption of the composite material (of 0.7% of the mass) took place by Day 11 and Day 55 for the 1 mm and 2 mm specimens, respectively. This would suggest that for thicker composites laminates, which would be typical in the structural components of tidal turbine blades, the time to reach maximum absorption would be significantly longer. However, this is still a low water absorption rate, compared to other materials, and a major advantage of using CPET. Additionally, for composite materials in operation, water ingress protection measures would be applied to the material, which were not used in this study.

The change in mechanical properties, which was observed within both the static and fatigue testing programmes, needs to be incorporated within the design of a marine composite structure. The most important material properties to incorporate are those in the 0° direction, which saw a reduction in tensile strength and tensile modulus of approximately 35% and 8%, respectively, after 182 days of conditioning in water at 50 °C. This is an important finding as failure will occur much earlier than the design life of the blade if these mechanical properties are not accounted for. Overall, this study shows that CPET is suitable for use in sub-marine environments as is has a low water absorption rate and excellent mechanical properties, which have been quantified through extensive static and fatigue qualification testing.

The advantage of using a curing approach instead of adhesive bonded joints has been demonstrated through lap shear coupon trials. The results from these tests show that this approach improves the strength of the joint by 61% on average (43.7 MPa compared to 27.1 MPa lap shear strength for an adhesive bond). Therefore, this technology is well suited to be applied to bond lines along the leading and trailing edge of the tidal turbine blade.

## Subcomponent Manufacturing Trials

### Thick Section Composite Manufacture

A major challenge is presented to the industry when producing thick section composite structures. Poor process control can lead to several problems, including incomplete ply consolidation, void formation, non-uniform cure, and difficulties in transferring exothermic heat out of the laminate. To minimise the risk involved, these aspects were examined when manufacturing thick section composite laminates.

#### Curing Trial

**Fig. 7:**
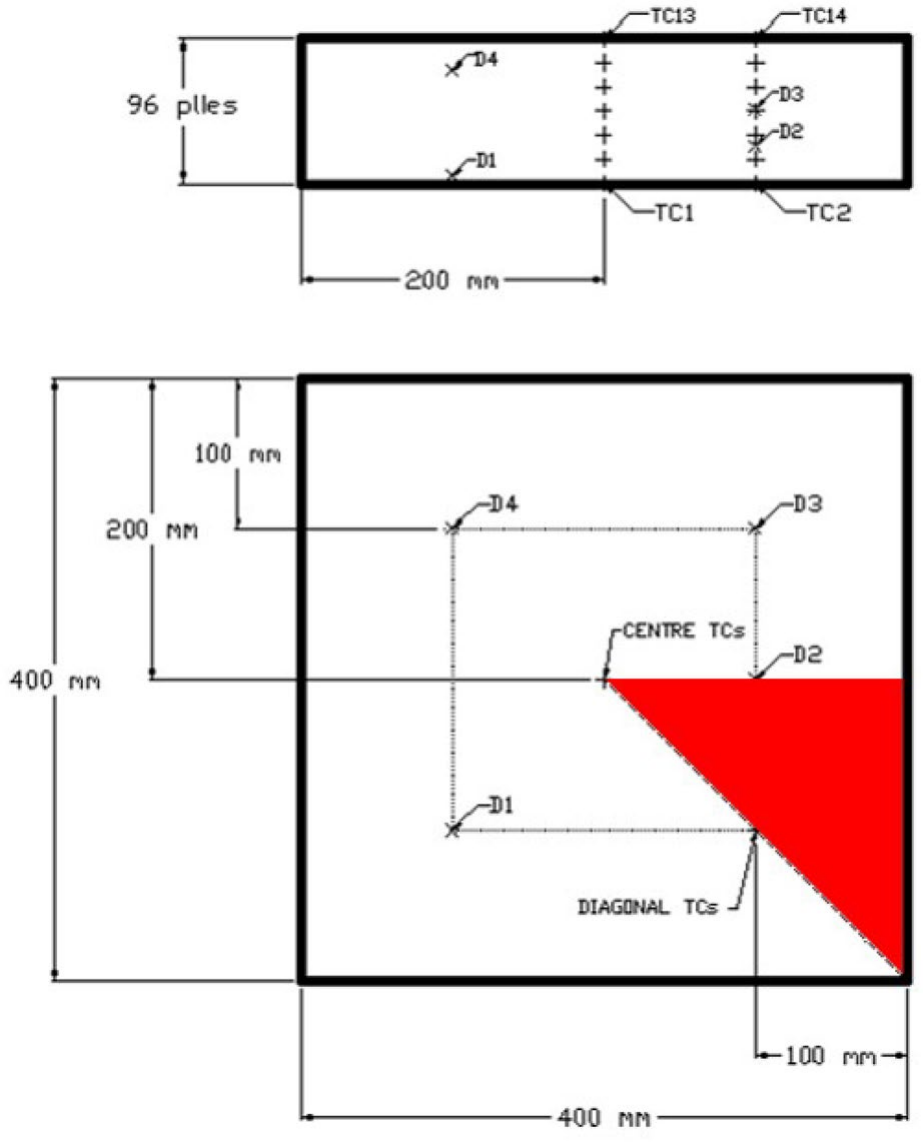
Schematic of the thick-section laminate with thermocouple (TC) positions identified. The red triangle highlights the region simulated in Abaqus FEA

**Fig. 8:**
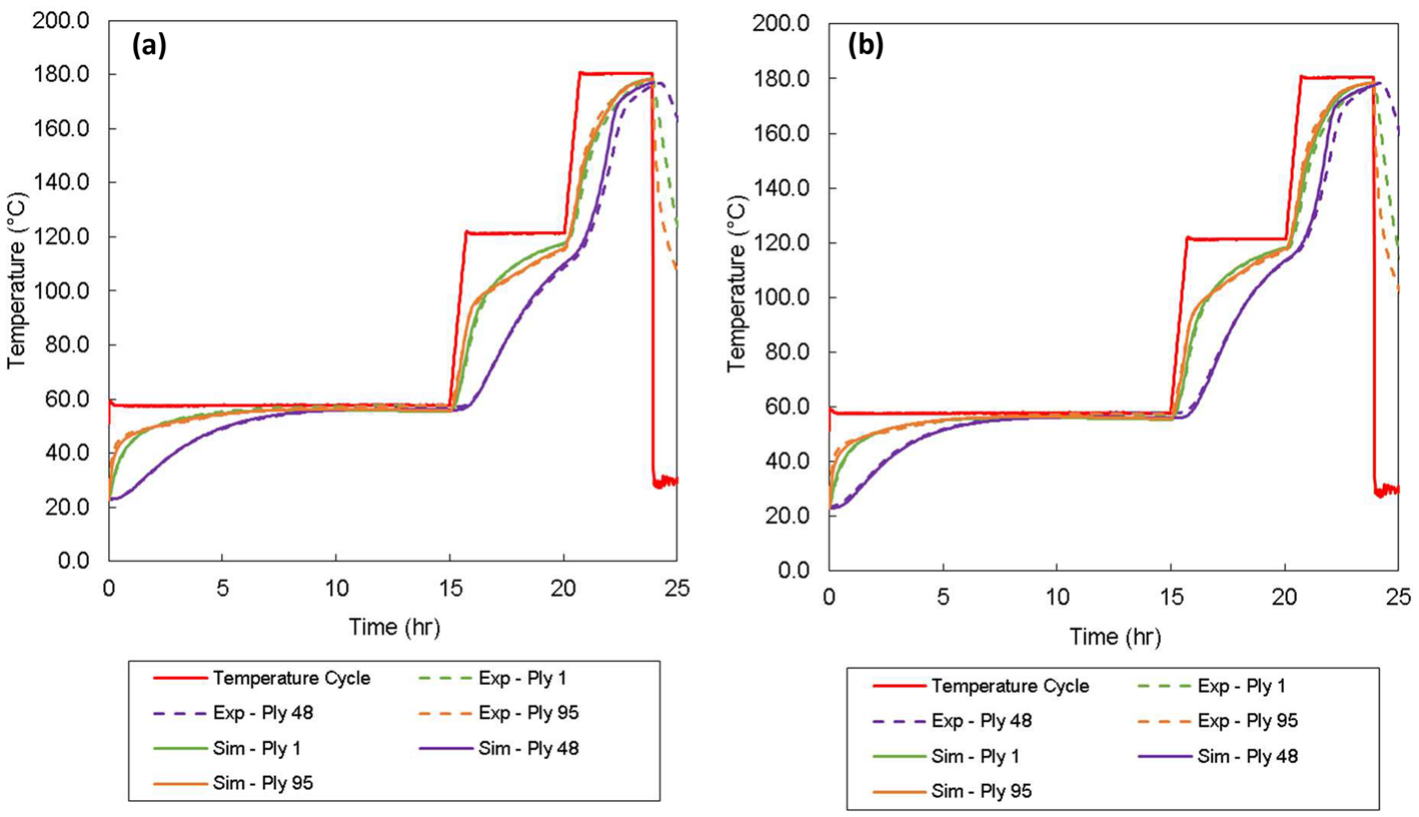
Comparison of the thermocouple data from the experimental trials and simulated temperatures at the corresponding locations from the numerical model (**a**) at the centre of the laminate and (**b**) towards the edge of the laminate

As an initial trial, a 96-ply glass-fibre/epoxy laminate was manufactured on a 10 mm thick flat steel tool in a fan-assisted oven. The laminate had in-plane dimensions of 400 × 400 mm and a cured thickness of approximately 96 mm. Thermocouples, which were embedded as shown in Fig. 7, were used to monitor the variation in temperature within the laminate. The trial showed that there was no thermal overshoot in temperature during processing and it was also determined that the exothermic heat within the laminate aided the curing process; see Fig. 8a and b.

**Fig. 9:**
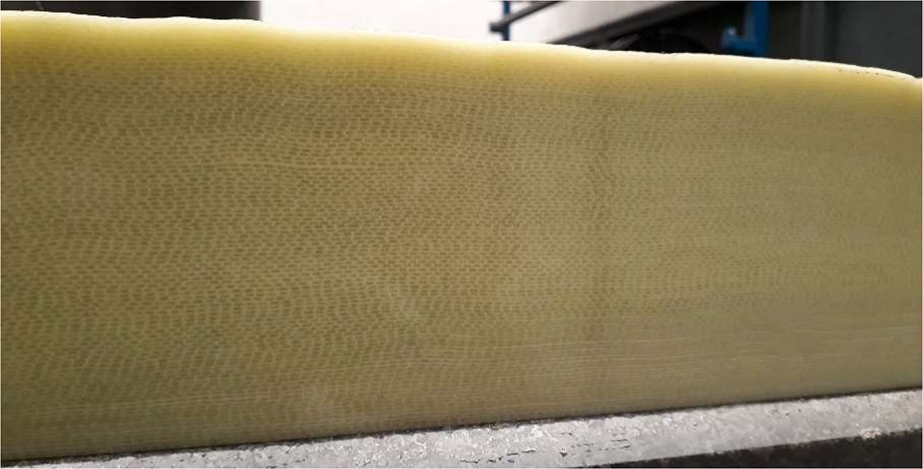
A cut section of the thick composite laminate that has been examined in this study

Once the curing trial was complete, the thick section laminate was cut in order to examine the cross-section through the depth of the composite laminate, which can be seen in Fig. 9. The main aspects that are being explored within this exercise are the ply consolidation and voiding within thick section composite structures. From Fig. 9, it can be seen that there was excellent consolidation with no voids visible. This gives great confidence in the manufacturing technique developed and its potential for commercial applications.

#### Numerical Modelling of Thick Laminates

Also shown in Fig. 8 are the results of numerical simulations. These results were produced using experimentally validated process models [9, 17, 22], which have been developed specifically for manufacturing fibre-reinforced composite parts using epoxy powder systems, and then implemented in Abaqus FEA [10].

**Fig. 10:**
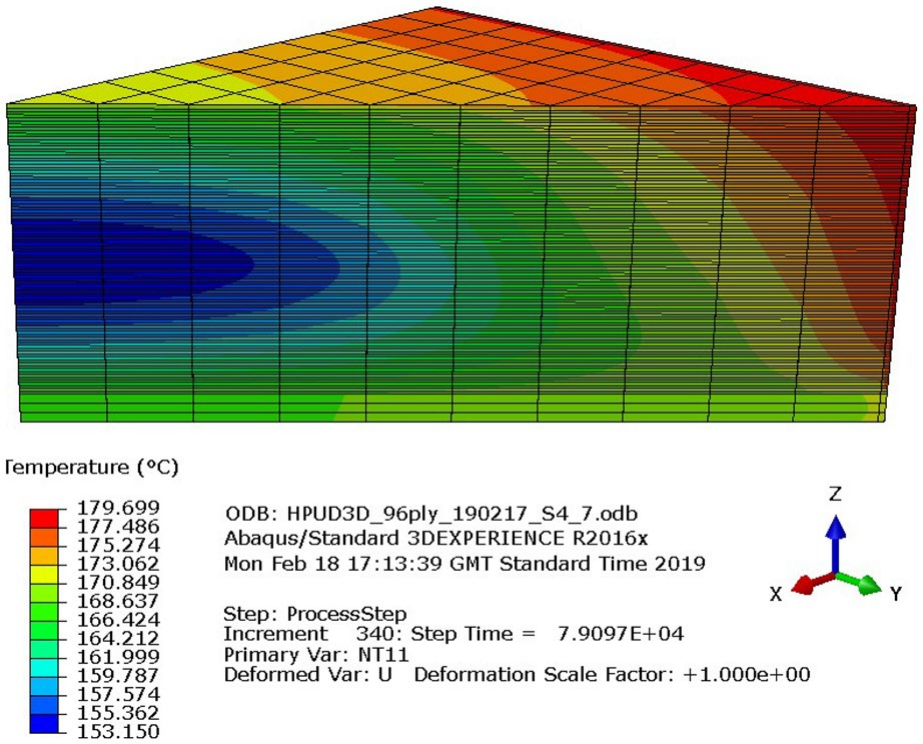
Three-dimensional visualisation of the process simulation in Abaqus FEA. The laminate is represented by a one-eighth slice (see Fig. 7). The contour plot shows the temperature variation in the laminate during the curing stage. Heat transfers from the outside of the laminate towards the centre

Numerical simulations were performed to ensure full consolidation and cure was achieved for a given processing cycle, thus reducing the amount of expensive trial and error experiments. The simulations were performed using fitted heat transfer coefficients for the thermal boundary conditions; 30 W/m^2^K and 25 W/m^2^K for the top and bottom side of the tooling, respectively. The agreement of the data showed that the simulation tool was robust and could be used to more efficiently investigate the processing of thick-section geometries, such as the root section of a tidal turbine blade. It was also possible to elucidate the temperature variation using a three-dimensional visualisation, as shown in Fig. 10.

The results of this analysis have practical implications in the efficient design of thick section composite structures processing, as the length of each curing stage can be optimised, while ensuring that the section is cured throughout.

#### Edge Manufacturing Trials

**Fig. 11:**
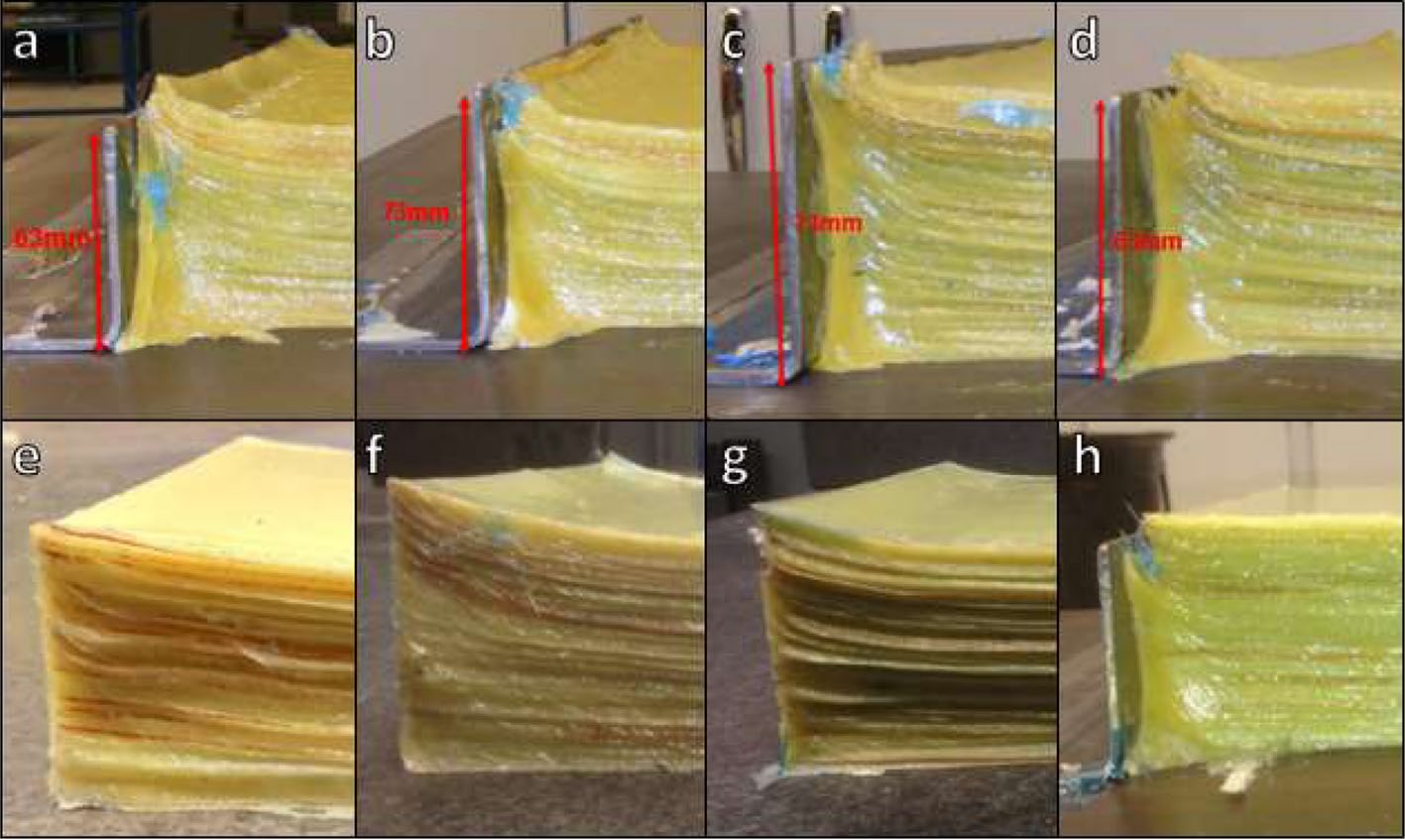
Edge manufacturing trials of thick section composite structures

An additional challenge that was presented while developing the manufacturing techniques for thick section composite structures was the production of evenly distributed, level laminates. Therefore, an experimental trial campaign was undertaken to investigate the best technique for addressing this challenge, the results of which are shown in Fig. 11. The following are the details of the experimental trials that took place:

- Trial a: End plate higher than the cured laminate thickness (63 mm), which was in contact with the layup
- Trial b: End plate same height as the cured laminate thickness (63 mm), which was in contact with layup
- Trial c: End plate was higher (73 mm) than the cured laminate thickness (63 mm), where there was a 2 mm gap between the layup and the end plate
- Trial d: End plate was the same height as the cured laminate thickness (63 mm), where there was a 2 mm gap between the layup and the end plate
- Trial e: End plate was higher than the de-bulked thickness (89 mm), which was in contact with the layup and a caul plate on top of laminate
- Trial f: End plate was higher than de-bulked thickness (89 mm), which was in contact with the layup, and a silicone sheet on top of layup, along with an 18 mm silicone intensifier
- Trial g: End plate was higher than de-bulked thickness (89 mm), which was in contact with the layup, and a silicone sheet on top of layup, along with a 36 mm silicone intensifier
- Trial h: End plate was same height as the cured laminate thickness (63 mm), which was in contact with layup, and a caul plate on top of laminate

The most successful trial was Trial h, which included both an end plate and a caul plate. This method will be developed further to de-risk the technique and, following this, it will be incorporated into the main manufacturing process for thick section composite parts, including its use in the production of tidal turbine blades.

### Blade Root Connection System

**Fig. 12:**
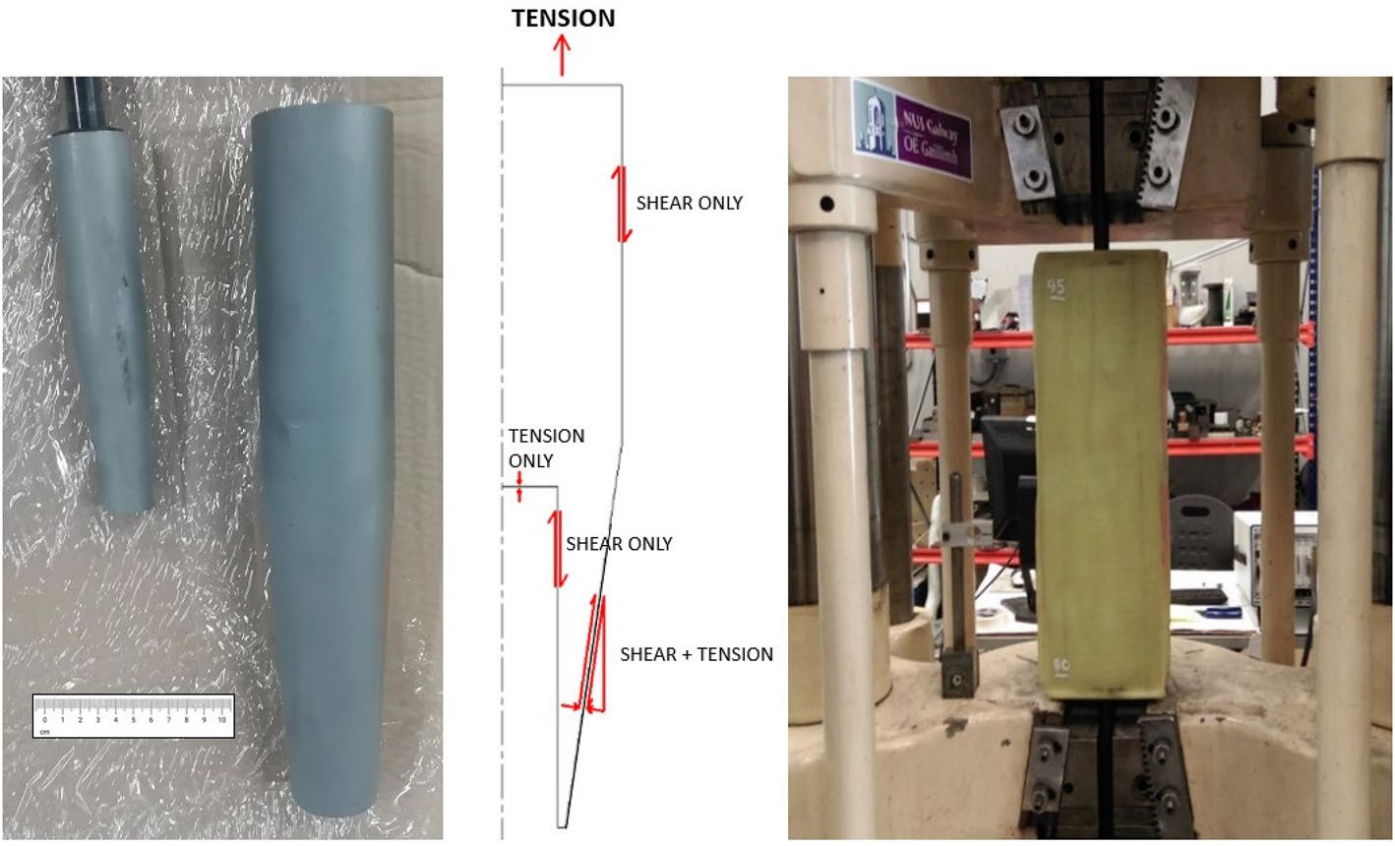
Root insert connections, showing (**a**) the $${\raise0.5ex\hbox{$\scriptstyle 3$}\kern-0.1em/\kern-0.15em\lower0.25ex\hbox{$\scriptstyle 8$}}$$ scale insert used for testing (left) and the full-scale insert (right), (**b**) the shear and tensile forces acting on the root insert-resin interface and (**c**) the test specimen during tensile testing

An efficient root connection system, using steel inserts with a high contact surface area, has been developed in order to connect the thick section composite blade root to the rotor hub. The root insert that will be used for the full-scale tidal turbine blade is shown on the right of Fig. 12a, where the $${\raise0.5ex\hbox{$\scriptstyle 3$}\kern-0.1em/\kern-0.15em\lower0.25ex\hbox{$\scriptstyle 8$}}$$ scale insert used for testing is also shown. A cross-section of the root insert, showing its geometry and the shear and tensile forces acting on the root insert-resin interface, which allows for the high contact surface area is shown in Fig. 12b.

#### Static Testing Programme

Initially, experimental trials were performed with a plain Sect. 20 mm steel bar co-cured in a thick section composite part in order to establish the maximum stress the bond could withstand, which resulted in a peak mean shear stress (MSS) on the composite-epoxy bond of 30 MPa.

Following this, a static tensile testing campaign was carried out on the steel inserts in order to assess the capacity of the root connections, which can be seen in Fig. 12c. In order to ensure that this stage of testing was within the capacity of the testing machine, $${\raise0.5ex\hbox{$\scriptstyle 3$}\kern-0.1em/\kern-0.15em\lower0.25ex\hbox{$\scriptstyle 8$}}$$ scale inserts (shown on the left of Fig. 12a) were used to assess the performance of this steel root insert design.

Two test specimens were tested in static tension and failed at loads of 315 kN and 334 kN, which represented a MSS at failure of 23 MPa and 24 MPa respectively. Therefore, based on these results, the static tensile capacity of the specimen (F_0_) is 324.5 kN, where the average MSS is 23.5 MPa. This was a drop off in MSS compared to the initial test with a steel bar, which was attributed to the additional design features that were incorporated to resist other load cases (i.e. bending moments and shear stresses). In both cases the failure occurred in the bolted connection rather than at the root insert-resin interface, which gives great confidence in the design.

#### Fatigue Testing Programme

**Fig. 13:**
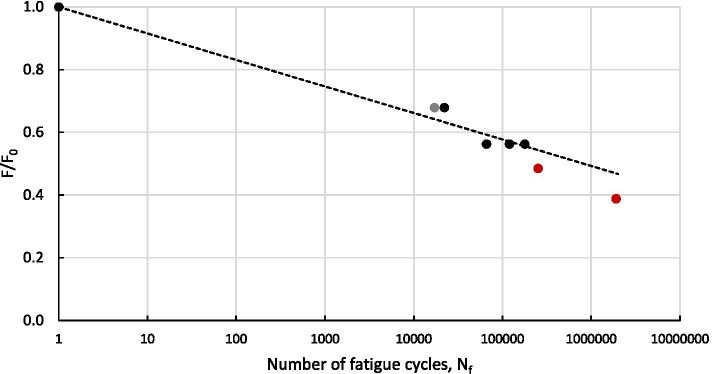
Summary of the fatigue tests based on their normalised loading with respect to the static testing results (F_0_) versus the number of fatigue cycles before failure, N_f_

A tension-tension fatigue testing campaign was completed on 7 root insert connection specimens using the tensile testing machine shown in Fig. 12c. These tests were performed in accordance to ASTM D3479/D3479M-19 [20], which is the standard test method for tension-tension fatigue of polymer matrix composite materials, and the results are summarised in Fig. 13. These tests were performed in order to ensure that the tidal blade can withstand the fatigue loading expected during operation [23].

Specimen failures occurred in two ways—a failure within the bond between the steel insert and the composite material or in the steel insert itself. For two of the test specimens, failure occurred in the steel insert itself, which are the red points in Fig. 13. The remainder of the specimens (5) failed as a result of a failure within the bond between the steel insert and the composite material. However, for one of the specimens at a normalised loading of 0.2F_0_, no failure was observed after 2,000,000 fatigue cycles. Subsequently, the normalised loading was raised to 0.68F_0_ and failure was observed after 17,109 fatigue cycles, which is represented by the grey point in Fig. 13. When designing the root inserts for tidal turbine, these normalised results can be used to predict the number of fatigue cycles to failure for a known loading, which can be incorporated into both design life prediction and structural health monitoring of the root connection of a tidal turbine blade in operation.

**Fig. 14:**
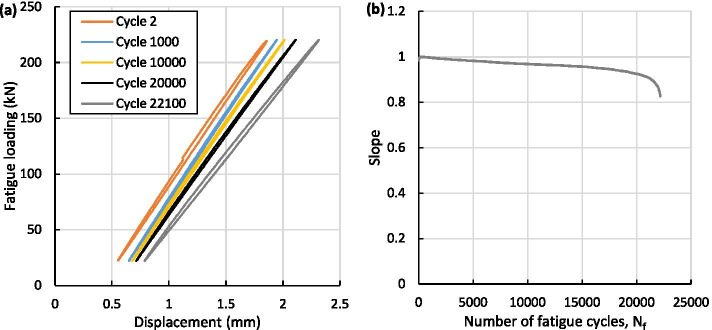
Results from the fatigue testing of a specimen at 0.68F_0_, showing (**a**) a sample selection of force–displacement hysteresis loops and (**b**) the variance in the force–displacement slope during the test

In order to demonstrate a further use of the data recorded during the fatigue testing of the specimens, the results from one of the specimens that was fatigue loaded to a maximum of 68% of the capacity achieved in the static tests (0.68F_0_), where the results are shown in Fig. 14. This specimen failed after 22,176 fatigue cycles and the results of this individual test is shown in Fig. 14. The load–displacement hysteresis loops during the fatigue test, for fatigue cycles 2, 1000, 10,000, 20,000 and 22,100, are given in Fig. 14a. The changes in mechanical properties of the specimen during the test can be seen in the variance of the shape of the hysteresis loops. As energy was dissipated in each cycle, due to both linear and non-linear deformations of the materials and connection to the root insert, the area of the hysteresis loops increased and moved gradually along the displacement axis. This suggested a softening of the materials and/or the connection to the root insert due to damage accumulation.

Figure 14b displays the change in slope of the hysteresis loops as the fatigue cycles increased, which was calculated at the points of maximum and minimum stress and was normalised by the initial value of the slope (i.e. the slope of the hysteresis loop for fatigue cycle 2). The slope reduces gradually throughout the test, suggesting that there was no major loss in stiffness or significant damage, which gave good confidence in the design and manufacture process used to produce the test specimen. In the final stages, the slope decreased rapidly, suggesting a debonding or connection loss between the composite material and the root insert, which was the ultimate failure in the specimen on inspection after the test was complete. During operation, strategically selected root inserts can be instrumented with strain gauges and monitored in order to predict failures in advance. This data could also be used to inform the structural health monitoring systems in place for the tidal turbine blades in operation.

## Full-scale blade manufacture

**Fig. 15:**
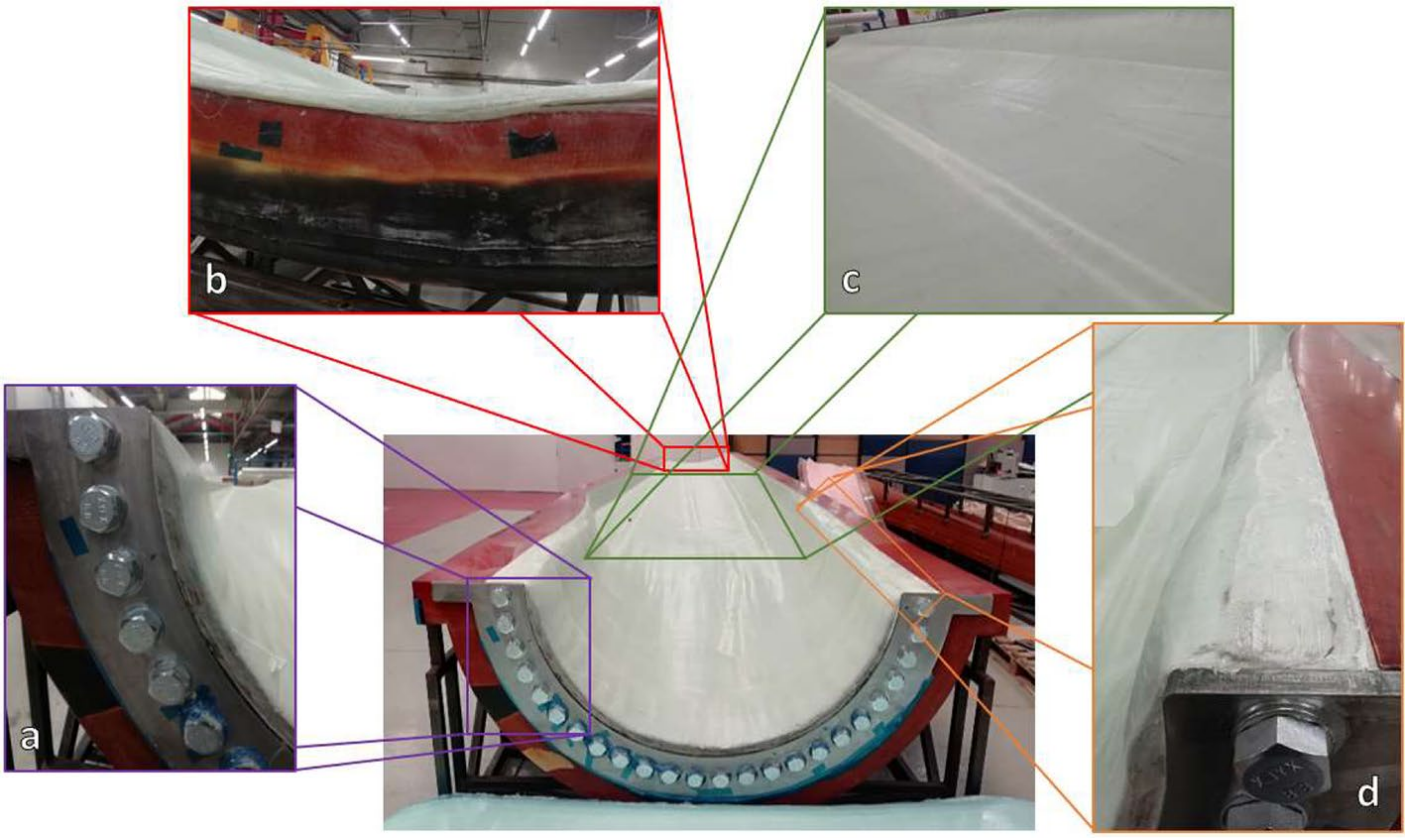
Graphical description of the main manufacturing challenges for the tidal turbine blade, showing (**a**) the root connection, (**b**) the thinner composite layup at the tip of the blade, (**c**) the spar caps built into the shell of the blade and (**d**) the thick composite layup incorporating the root connection and tapering away towards the tip

The tidal blade manufactured in this paper uses a one-shot manufacturing process, which is discussed in Sect. 5.1. The advanced manufacturing technologies that have been developed previously in this paper have been incorporated into a full-scale tidal turbine blade, where the key advances can be seen for one half of the blade in Fig. 15 and are discussed in Sect. 5.2. The composite material layup and individual component manufacture is also discussed in Sect. 5.3 and Sect. 5.4, respectively.

### One-Shot Manufacturing Process

Unlike traditional glass fibre epoxy manufacture, where exotherm from the resin during cure must be controlled to avoid damage to the part or a potential health and safety hazard [24], there is no significant exotherm during the one-shot manufacturing process for CPET [16], which has been discussed in Sect. 2.3. The one-shot manufacturing process for CPET was introduced in [16, 25] and details for how it has been applied for the manufacture of a large tidal blade are given in this section.

The material is solid at room temperature but is formable, without polymerisation, at low temperatures. This means that individual sub-structure components can be formed off-line on low cost, low temperature tooling and assembled to form the final part, which is then polymerised during the one-shot cure. A distinct advantage is this process enables the manufacture of large parts with complex internal structures without secondary bonding procedures. Therefore, eliminating the need for gluing along the bond line, which is further reinforced by overlapping plies, resulting in a stronger bond when compared to traditional manufacturing techniques, which is evident from the results from the lap shear testing presented in Sect. 3.4. Additionally, it enables joints with complex ply drop-offs and overlaps that would be difficult or impossible with traditional bonding techniques. CPET forms a bond to metal during cure which allows metal inserts to be embedded in the layup during cure, eliminating the need to drill and glue inserts. Furthermore, there are no volatile organic compounds emitted during cure, which means that the manufacturing process is in line with the tightest European manufacturing regulations [26]. The material is supplied in a semi-pre-impregnated form, where the glass is uniformly coated in powder epoxy. This means that during cure the powder epoxy only has to travel through the thickness of one ply in order to ensure that the laminates are fully wet-out and that there is uniform fibre volume throughout the entire structure.

### Integration of Advanced Manufacturing Techniques

Thick section laminates have been used to manufacture the spar caps, which are integrated into the skin of the blade, which can be seen in Fig. 15c. Additionally, thick section laminates have been used in conjunction with the innovative root connection system to form the root connection of the blade, which will support the blade and transfer the loading to the turbine hull, which can be seen in Fig. 15a and d, where the thickness of the composite in in excess of 125 mm.

It is vital for the root section of the blade to be much thicker than the remainder of the blade as it requires robust sections with the capacity to withstand the mechanical and structural loads imposed on the tidal turbine blade over its operation design life. The main purpose of the outer profile of the blade is to achieve efficient hydrodynamic performance in the presence of the incoming flow velocity. Toward the tip of the blade the laminates can be much thinner (approximately 15 mm in thickness), which can be seen in Fig. 15b.

### Composite Material Layup

**Fig. 16:**
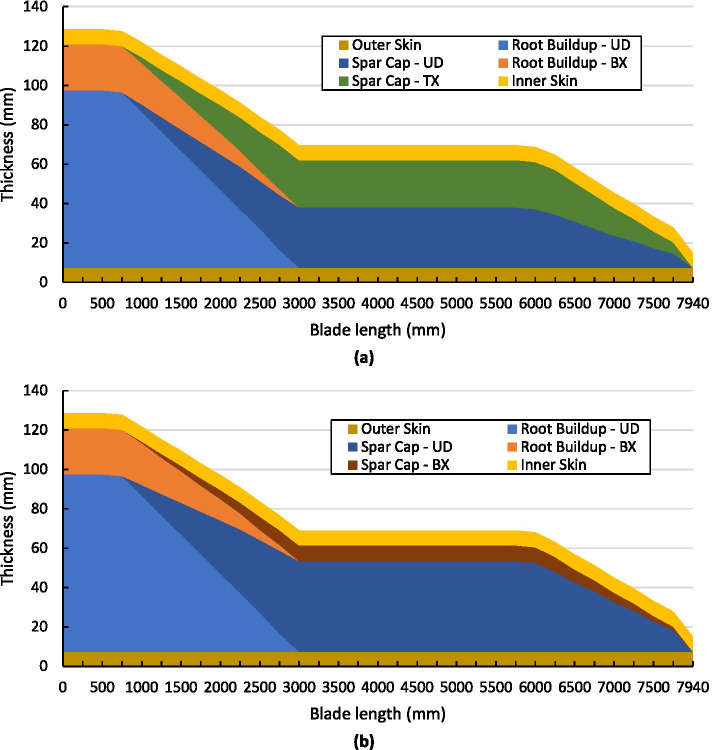
Simplified summary of the composite layup and its thickness (in mm) along the length of the tidal blade (in mm), where (**a**) is the lower section and (**b**) is the upper section

The features discussed in Sect. 5.2 are incorporated into the upper and lower skin section sub-structure components during the composite layup on the tooling. A combination of unidirectional (UD) and triaxial (TX) and biaxial (BX) composite material plies are used to form, both, the composite root section and the composite spar caps section of the blade. A summary of the composite layup and its thickness along the length of the tidal blade for the upper and lower skin sections is presented in Fig. 16. From this figure, the thicker root section is illustrated with ply drop-offs of the material towards the tip, as specified in the tidal turbine blade design.

### Component Manufacturing Processes

Before final assembly using the one-shot manufacturing process, which was described in Sect. 5.1, pre-forms of sub-structure components of the tidal turbine blade were laid up and “b-staged”. The tidal turbine blade was split into three main pre-forms. These were a upper and lower section preforms, which each included the root build-up, spar cap and skin plies, as well as the shear web preform.

**Fig. 17:**
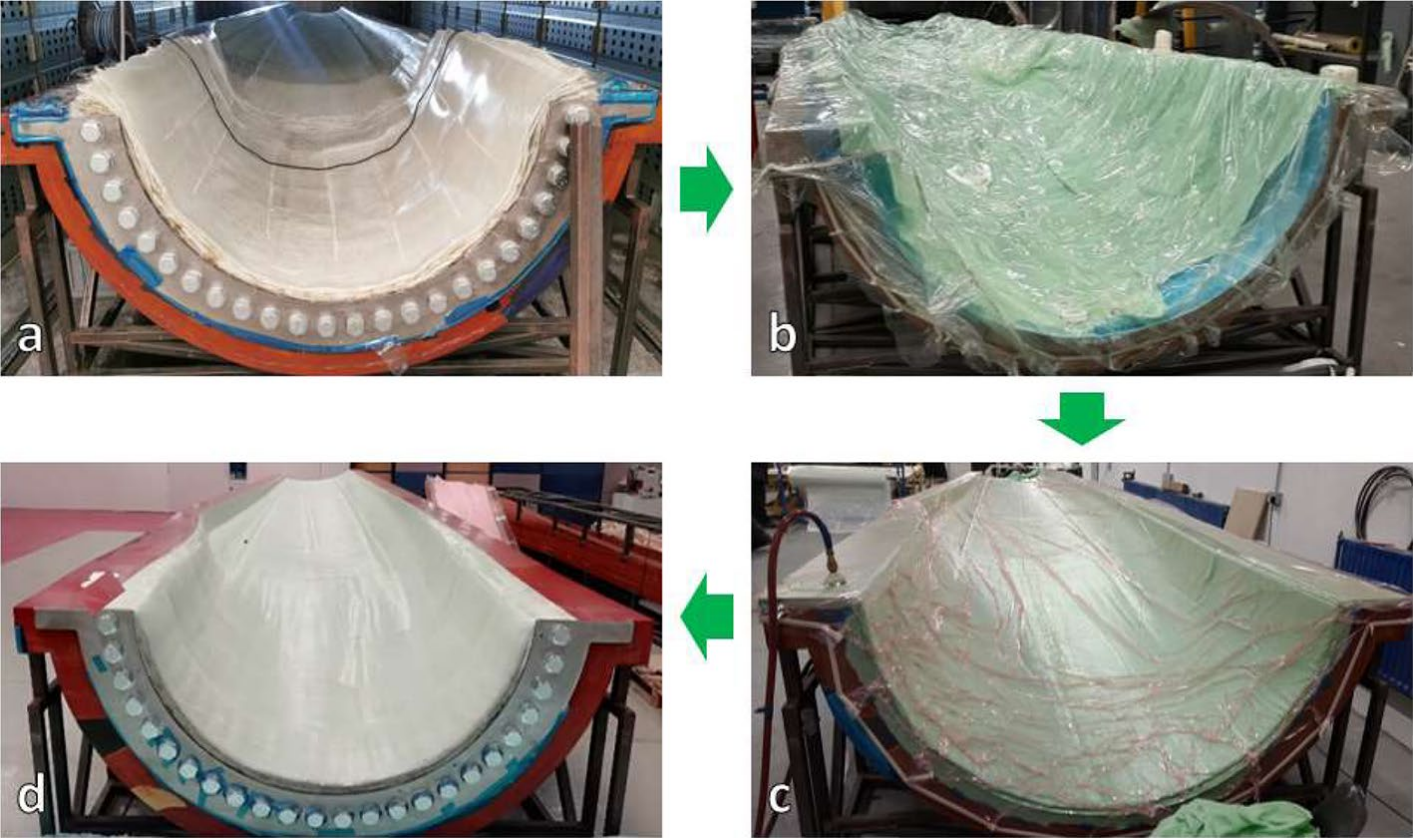
Stages of manufacture of the sub-components, detailing (**a**) ply layup on the tooling, (**b**) breather membrane and vacuum bag in place on the ply layup, (**c**) ply layup consolidated using vacuum and (**d**) final sub-component after b-stage cure under vacuum

The manufacturing processes for each of the preforms are broken down into four stages, which are illustrated in Fig. 17, and are as follows:

1. The composite material layup on the tooling is performed in a number of stages, depending on the desired final thickness of the component, which can be seen for the final 8 plies being laid up on a skin section sub-structure component in Fig. 17a.
2. Once the stage of layup is complete, the breather membrane and vacuum bag are placed on top of the ply layup, as seen in Fig. 17b. The vacuum bag is sealed to the tooling with a high-temperature double-sided adhesive tape.
3. A vacuum fitting is then fitted in the vacuum bag and connected to vacuum pump, this is seen in Fig. 17c. A minimum vacuum of 850 mbar is applied to consolidate the composite material layup during the cure.
4. The composite material layup is then place in an oven for a b-stage cure under vacuum, which is performed at 120 °C with a step at 55 °C.

These stages are repeated until all of the composite material has been laid up for the sub-structure component. An example of the finished skin section sub-structure component is shown in Fig. 17d.

**Fig. 18:**
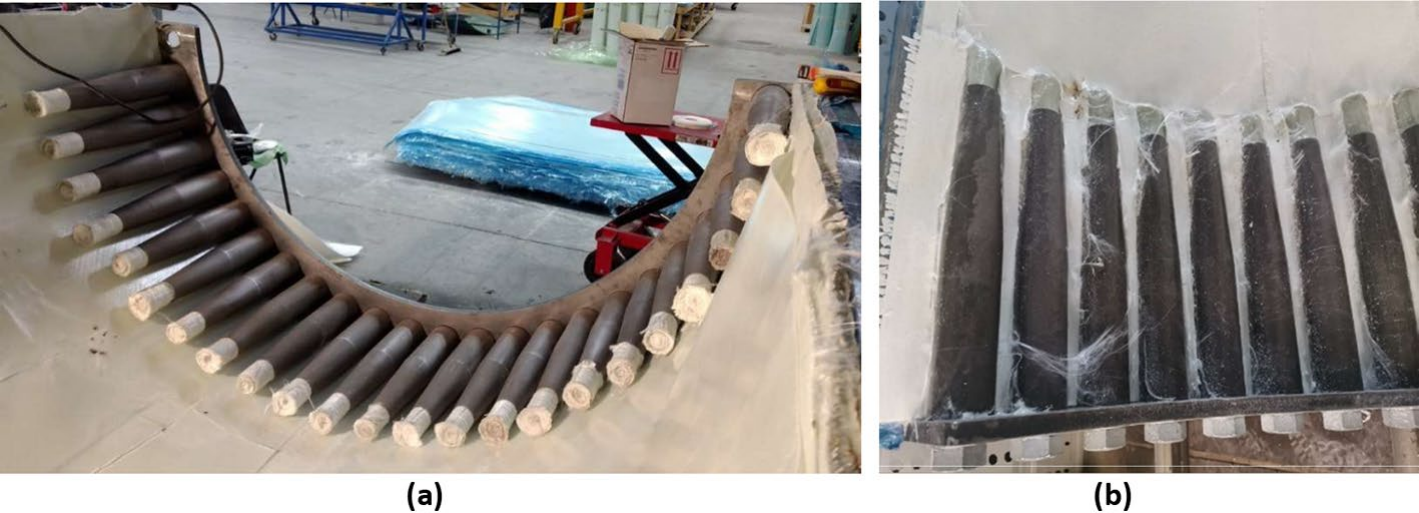
Detail of installation of the steel root inserts during skin section manufacture, showing (**a**) the steel root inserts after initial installation and (**b**) the composite ply layup around the steel root inserts

The complex ply layup around the steel root inserts, which connect the blade to the turbine hub, are shown in Fig. 18. This involves using relief cuts in the CPET material to install it around the steel root inserts and separate UD CPET material is placed inside the end of the steel root inserts. This will cause the powder epoxy to bond with the steel root inserts when it is cured, allowing the frictional stress within this bond to keep the steel root inserts in place during turbine operation.

### Final Tidal Turbine Blade Assembly

**Fig. 19:**
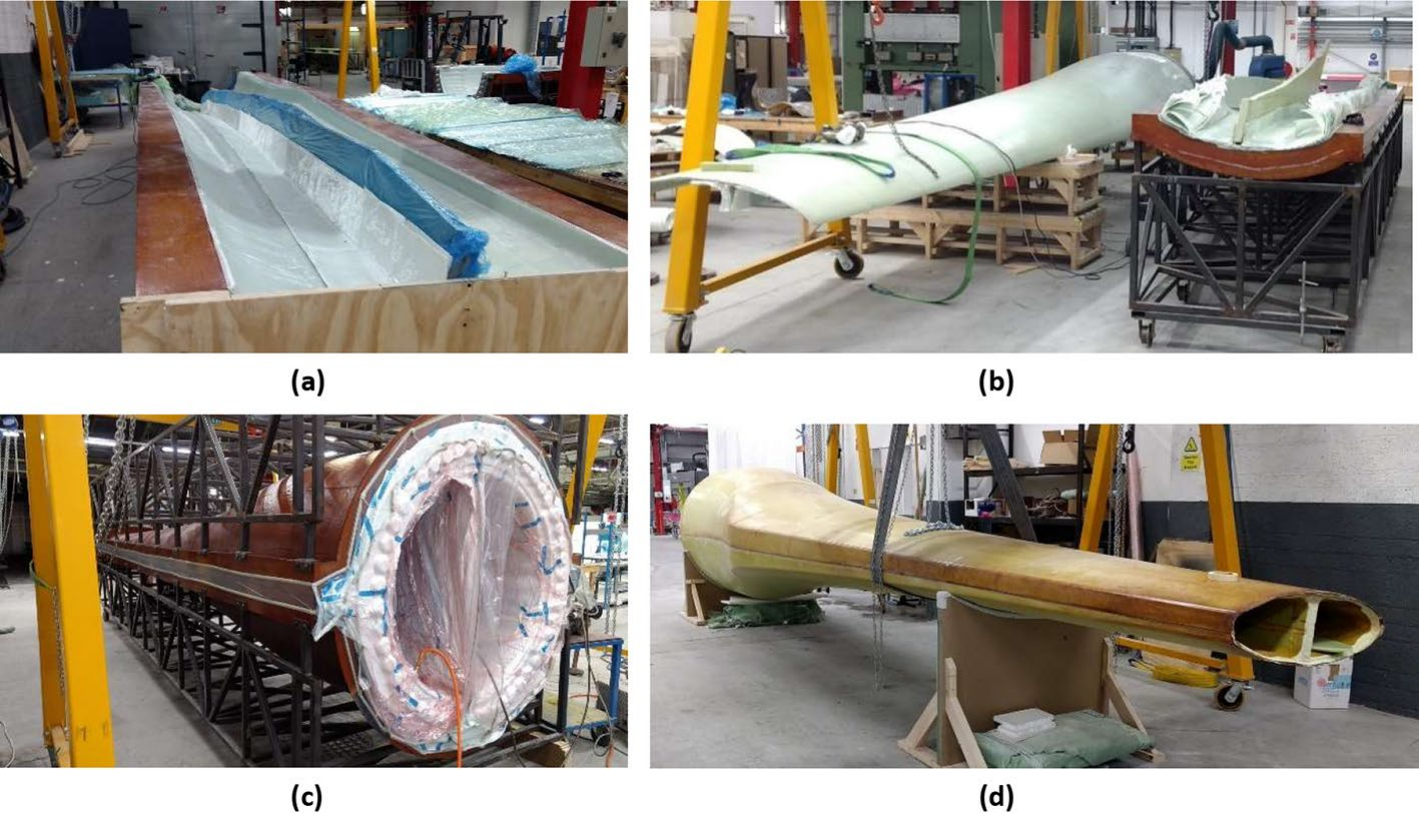
Tidal turbine blade assembly process, showing (**a**) the channel formed for the shear web on the spar caps, (**b**) the shear web in place on the lower skin section sub-structure, (**c**) the final assembly vacuum bagged in place before the final cure and (**d**) the blade after being demoulded

Once each of the three sub-components of the tidal turbine blade have been manufactured—upper and lower section preforms and the shear web preform – the next stage of manufacture is the final assembly. In this stage, the three sub-components are assembled and cured under vacuum at 185 °C. The steps required for this final stage of manufacture are described in detail, as follows:

1. A channel was formed for the shear web in both the top and bottom shell halves. This channel consisted of biaxial glass fibre plies and serves both to position the shear web for assembly as well as to tie the shear web to each shell half in the finished assembly. The channel was laid up against temporary tooling, shown in Fig. 19a and “b-staged” using the process described in Sect. 5.4.
2. The temporary tooling was removed, and the shear web was positioned into the bottom shell half, shown in Fig. 19b. Overlap plies were placed at the leading and trailing edge of the bottom shell half, these serve to tie the two shell halves together in the final assembly and reinforce the bond lines between the each shell half. The top shell half was then lowered in placed over the bottom shell half and shear web, completing the assembly.
3. The tooling for the top shell half was placed. The assembly was then vacuum bagged like as described in Sect. 5.4. This is shown in Fig. 19c. The pressure applied by the vacuum bag consolidates the three pre-forms as well as the overlap plies during the cure.
4. The assembly was moved to an oven and fully cured under vacuum. The full cure is performed at 185 °C with a step at 55 °C and 120 °C.
5. Once cured the assembly was removed from the oven and demoulded. The cured turbine blade is shown in Fig. 19d.

For tidal turbine blades that are to be used in operation, another step of the process will be performed to fill the cavity in the blade with a material with a similar density to that of water and apply a surface finish to the blade to help prevent water ingress during operation. However, the next stage of development for this tidal turbine blades will be full-scale structural testing, where strain and displacement sensors will be mounted on the blade substrate material (on the outer surface and along the shear web). Therefore, no surface finish or cavity fill have been performed on the blade.

## Discussions

The production of a composite tidal turbine blade presents a number of technical challenges in order to withstand the environment it will be deployed in and the high loads imparted on the blade during operation.

An extensive experimental investigation on the effect of water ingress on the CPET material has been performed in Sect. 3 to evaluate any knockdown in material properties, which can then be accounted for in the design of the full-scale composite tidal turbine blade. Specimens were saturated in water at 50 °C for up to 183 days, which is the equivalent submerged operational time of approximately 5 years, and then static and fatigue tested. An investigation into water absorption of CPET specimens was also performed but, as the samples were either 1 mm or 2 mm in thickness, further trials of samples of at least 30 mm in thickness are advised in order to understand water absorption and water ingress for thick section laminates, which are required to withstand the high loadings of tidal turbine blades. A change in mechanical properties was observed within both the static and fatigue testing programmes, where the most important material properties to incorporate are those in the 0° direction, which saw a reduction in tensile strength and tensile modulus of approximately 35% and 8%, respectively, after 182 days of conditioning in water at 50 °C. This is an important finding as failure will occur much earlier than the design life of the blade if these mechanical properties are not accounted for. This information was then used in the design of the full-scale composite tidal turbine blade.

The thickness of the composite material along the spar caps and at the root of the blade is significantly thicker than that seen for most composites applications, where the composite was 130 mm in thickness at some locations. Therefore, it was necessary to design bespoke curing cycles, which included numerical modelling with experimental validation, and manufacturing trials along with edges of these thick sections, where it was found that the use of a caul plate with a height of the expected finish thickness of the section was the desired approach. The manufacture of the tidal turbine blade, in Sect. 5, was the full-scale application of these techniques and no issues with thickness were observed during manufacture. It is anticipated that an investigation of the thick-section elements will be performed once the full structural testing (in both static, for the maximum expected load, and fatigue, over the design life of 20 years) is completed.

The high loads imparted on the blade during operation require a robust root connection system – therefore, steel inserts with a high contact surface area were designed, manufactured and installed in the blade in this study. Mechanical testing was performed on the steel inserts to ensure they could withstand the expected loading. However, a further mechanical investigation of this system for a composite that has been conditioned, similar to the studies performed in Sect. 3, would yield more information about their performance during operation. It was observed that more time is required for the installation of these steel inserts during manufacture, which is discussed in Sect. 5.4, but this is offset, in part, by not needing to install a root connection system post cure, which provides a highly robust connection.

One of the advantages of using the CPET composite material is that a curing approach instead of adhesive bonded joints can be employed. The effectiveness of this was explored through lap shear coupon trials, where cured bonded joints improved the strength of the joint by 61% on average (43.7 MPa compared to 27.1 MPa lap shear strength for an adhesive bond). This study gives great confidence in this approach and shows the viability of using a more efficient approach, in terms of manufacture, than the traditional techniques. Therefore, this technique was used along the leading and trailing edge of the tidal turbine blade, as discussed in Sect. 5.

The composites manufacturing technologies that are developed and discussed in this paper will aid in producing tidal turbine blades that can survive their harsh operational environments over their design life. It is acknowledged in this section that there still are aspects that need further development and investigation, along with the lessons learned from the study, but the full-scale application of these technologies have been successfully applied for a large tidal turbine blade.

## Conclusion

In this paper, a range of advanced manufacturing technologies for producing a blade for a 1 MW tidal turbine rotor are developed, including thick section laminate manufacture for the root and spar caps and an efficient root connection system. Additionally, the effect of saturated conditions on the composite material properties and strength along bonded joints are explored. This paper presented a large case study of an 8-m tidal turbine blade, where the challenges relating to thick composite sections at the root and along the spar caps (> 130 mm in places), the fast changes in geometry over a short length that isn’t the case for wind blades and the required durability of the material in the marine environment had been overcome. The results show that CPET is a suitable material for parts operating in harsh marine environments, such as wind and tidal energy as well as boats. The manufacturing processes described in this study will significantly progress the manufacture of tidal turbine blades, while improving their reliability in operation.

In order to prove these new technologies, the tidal turbine blade will go through a rigorous testing programme. Initially, mechanical static, dynamic and fatigue loadings will be applied to the blade, followed by in-service operational sea trials. During the structural testing, an accelerated fatigue testing programme will take place where an equivalent loading of that experienced over 20 years of operation will be imposed on the blade. Therefore, demonstrating the performance of the materials, manufacture processes, blade design and the blade itself over its design life. It is expected that the manufacturing technologies developed in this study will reduce the labour requirement for producing tidal turbine blades. For small production runs, the initial tooling cost is significant. However, for volume production, labour is the most significant cost and using these technologies will result in a cost reduction of approximately 20% during production.

Therefore, the major impact of this study will be to both lower the levelised cost of tidal energy and increase the design life of tidal turbine blades to over 20 years by ensuring that tidal turbine blades have the best chance of surviving the harsh environment in which they are deployed. The longevity of the blades and their ability to withstand high mechanical loadings, both static and fatigue, throughout their design life span will significantly reduce maintenance costs and allow for uninterrupted energy generation, therefore aiding to lower the levelised cost of tidal energy.

## Data Availability

Publicly available testing data available in this publication. Additional testing data may be granted through contact with William Finnegan.

## List of Notation

RTM: Resin transfer moulding
VARTM: Vacuum assisted resin transfer/infusion moulding
CPET: ÉireComposites’ Composites Powder Epoxy Technology
FEA: Finite element analysis
MSS: Mean shear stress
F_0_: Static tensile capacity of the specimen
UD: Unidirectional composite material plies
TX: Triaxial composite material plies
BX: Biaxial composite material plies
